# Over-expression of CKS1B activates both MEK/ERK and JAK/STAT3 signaling pathways and promotes myeloma cell drug-resistance

**DOI:** 10.18632/oncotarget.105

**Published:** 2010-05-15

**Authors:** Lei Shi, Siqing Wang, Maurizio Zangari, Hongwei Xu, Thai M. Cao, Chunjiao Xu, Yong Wu, Fang Xiao, Yinghong Liu, Ye Yang, Mohamed Salama, Guiyuan Li, Guido Tricot, Fenghuang Zhan

**Affiliations:** ^1^ Division of Hematology/BMT/myeloma Program, University of Utah School of Medicine, University of Utah, Salt Lake City, UT,USA; ^2^ Huntsman Cancer Institute, University of Utah, Salt Lake City, UT, USA; ^3^ Cancer Research Institute, Central South University, Changsha, China; ^4^ The Second Affiliated Hospital of Xiang Ya School of Medicine, Central South University, Changsha, China; ^5^ Department of Pathology, ARUP Reference Laboratory, University of Utah, Salt Lake City, UT, USA

**Keywords:** Myeloma, CKS1B, ERK1/2, STAT3, drug resistance

## Abstract

Here we demonstrate the crucial role of CKS1B in multiple myeloma (MM) progression and define CKS1B-mediated SKP2/p27^Kip1^-independent down-stream signaling pathways. Forced-expression of CKS1B in MM cells increased cell multidrug-resistance. CKS1B activates STAT3 and MEK/ERK pathways. In contrast, SKP2 knockdown or p27^Kip1^ over-expression resulted in activation of the STAT3 and MEK/ERK pathways. Further investigations showed that BCL2 is a downstream target of MEK/ERK signaling. Stimulation of STAT3 and MEK/ERK signaling pathways partially abrogated CKS1B knockdown induced MM cell death and growth inhibition. Targeting STAT3 and MEK/ ERK signaling pathways by specific inhibitors induced significant MM cell death and growth inhibition in CKS1B-overexpressing MM cells and their combinations resulted in synergy. Thus, our findings provide a rationale for targeting STAT3 and MEK/ERK/ BCL2 signaling in aggressive CKS1B-overexpressing MM.

## INTRODUCTION

Multiple myeloma (MM) is a plasma cell malignancy, which remains largely incurable with current therapeutic strategies. The molecular bases of MM progression and drug-resistance are not completely understood. Gain of chromosome 1q21 is one of the most recurrent chromosomal aberrations observed in MM and correlates with disease progression [1]. We previously identified a 70 high-risk signature pattern in MM, with over-representation of over-expressed genes mapping to chromosome 1q, further supporting the hypothesis that 1q21 gain may be important in MM progression. High expression of *CKS1B*, a Cdc28 protein kinase regulatory subunit 1B, mapping to the 1q21 amplicon [2] and one of the 70 high-risk signature gene pattern, has been shown to be inversely related with survival in MM [3]. Additionally, knockdown of CKS1B in MM cells potently induced growth inhibition and apoptosis, suggesting that CKS1B plays a crucial role in MM cell survival [4]. However, the functional role of CKS1B in MM cell survival and MM disease progression remains to be elucidated.

CKS1B has a well-documented role in the cell-cycle regulation. Cks1 is a member of the Cks/Suc1 family of proteins, which are essential components of cyclin-dependent kinases (CDKs) that regulate mitosis in all eukaryotes [5]. CKS1B was identified as an essential accessory protein to the SCF^SKP2-CKS1^ ubiquitin ligase complex [6-8]. In this complex, Cks1 enhances the interaction between SKP2 and p27^Kip1^ (a CDK inhibitor), resulting in cell proliferation [9]. The role of Cks1 in regulating p27^Kip1^ proteasome degradation in the cell cycle is particularly evident in CKS1–/– mice. The key feature of these mice is their small body size, which is due to lack of cell proliferation, secondary due to lack of degradation of the G1-/S-phase CDK inhibitor p27^Kip1^[6]. Interestingly, besides influencing cell growth and survival through regulation of p27^Kip1^, silencing of CKS1B also induces cell death and inhibits growth in MM cells in the presence of a bi-allelic deletion of the *CDKN1B* (p27^Kip1^) locus, indicating that CKS1B mediates its effects on cell growth and survival also through mechanisms that are independent p27^Kip1^ and SKP2 [4].

In this study, we found that forced expression of CKS1B by lentivirus vector-mediated CKS1B-cDNA transfection in MM cells increased drug-resistance, providing direct evidence of the crucial role of CKS1B in MM progression. Furthermore, we also identified STAT3 and MEK/ERK/ BCL2 pathways to be downstream targets of CKS1B activation independent on the complex of SKP2/p27^Kip1^.

## RESULTS

### CKS1B expression is increased in relapsed MM and confers a short post-relapse survival

**Fig. 1:**
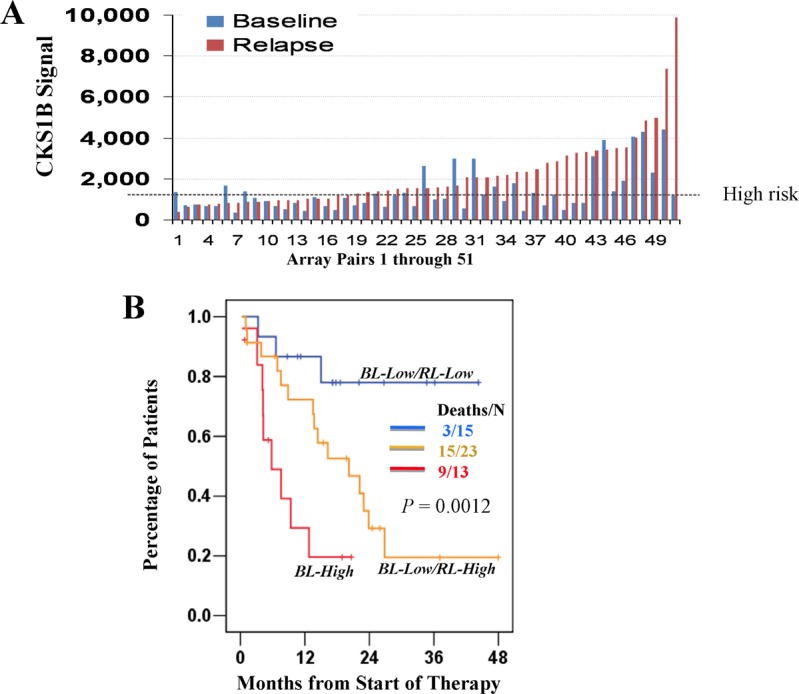
Increased CKS1B expression in relapsed myeloma links a short postrelapse survival **(A)** CKS1B signal for 51 paired arrays was obtained at diagnosis and relapse. The high risk (quartile 4) reference line is taken from the complete (n=351) sample of arrays at diagnosis. Note that a majority of samples showed increased expression at relapse; the most dramatic changes were observed in patients with expression levels in quartiles 1–3 at diagnosis. A paired Student *t* test was used to compare log-scale signal at diagnosis and relapse. **(B)** Kaplan-Meier analysis of postrelapse survival is shown in relation to *CKS1B* expression from low expression at baseline (BL-Low) to low expression at relapse (RL-Low; n = 15) and BL-Low to high expression at relapse (RL-High; n = 23) and already high expression at baseline (BL-High; n = 13) determined by microarray. At the time of analysis, the median follow-up of a post-relapse survival was 14 months (range, 0.3 to 50 months) in this analysis..

Our previous studies showed that CKS1B was one of the 70 high-risk genes, inversely associated with survival in newly diagnosed MM [3]. We compared CKS1B expression in 51 patients with paired baseline (diagnostic) and relapse samples. The median signals of CKS1B from microarray data at diagnosis and at relapse were 1398 (range: 370 ~ 4433) and 2174 (range: 405 ~ 9867), respectively. *CKS1B* expression increased in 76% of relapsed MMs and was more than 1.5 fold higher in 51% (Figure 1A; *p* = 2.39× 10^−5^).

As we expected, patients, who had CKS1B expression in quartile 4 (high-risk) at baseline and receiving various salvage therapies had the worst 4-year post-relapse survival (Figure 1B; *p* = 0.0012). The quartile 4 reference line is taken from the complete sample (n= 351) of arrays at diagnosis [3, 10]. Interestingly, among 38/51 relapsed patients with low CKS1B expression (quartiles 1 ~ 3) at baseline, but who showed increased CKS1B expression of at least 1.5 fold at relapse had inferior 4-year post-relapse survival compared with those lacking a 1.5 fold CKS1B up-regulation at relapse (Figure 1B; *p* = 0.032). Furthermore, among 36 relapsed patients with high CKS1B expression at relapse, the 4-year post-relapse survival of those with high CKS1B at baseline and at relapse was significantly worse compared with that of patients with high CKS1B expression only at relapse (Figure 1B; *p* = 0.0247). These data further confirm that *CKS1B* expression is a prognositic marker especially at diagnosis, but also at relapse.

### CKS1B over-expression promotes MM cell drug-resistance

**Fig. 2:**
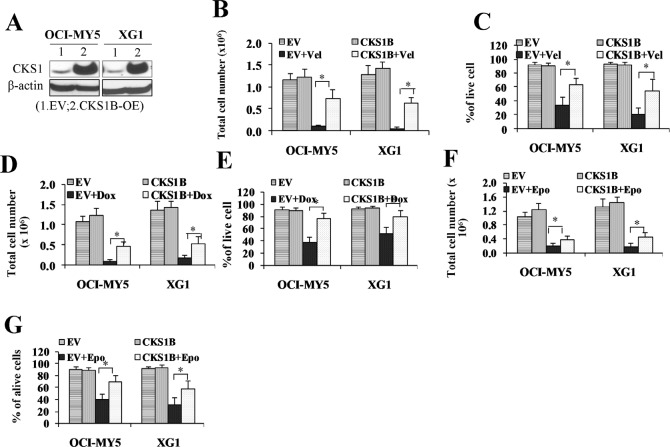
CKS1B over-expression promotes myeloma cell drug-resistance **(A)** Western blots examined CKS1B proteins increased in CKS1B-transfected OCI-MY5 and XG-1 cells (CKS1B-OE). **(B - G)** MM cells overexpressed CKS1B showed multi-drug resistance. CKS1B-over-expressing OCI-MY5 and XG-1 cells were treated with bortezomib (Vel; 5 nM), doxorubicin (Dox; 100 nM), and etoposide (Epo; 100 nM) for 48 hours in the cultures, and cell growth (B, OCI-MY5: 0.11M vs. 0.75M, and XG1: 0.06M vs. 0.65M; D, OCI:-MY5: 0.11M vs. 0.47M, and XG1: 0.19M vs. 0.54M; F, OCI-MY5: 0.21 vs. 0.39M, and XG1: 0.19 vs. 0.47M) and death (C,OCI-MY5: 34% vs. 63%, and XG1: 20% vs. 54%; E, OCI-MY5: 38% vs. 77%, and XG1: 53% vs. 81%; G, OCI-MY5: 41% vs. 71%, and XG1: 32% vs. 59%) were evaluated for these three treatments respectively. Untreated and EV cells with or without drug treatment were used as controls. Results were expressed as Mean+SD of three independent experiments (*p <0 .05).

Increased expression of CKS1B is a progression event, but it is possible that CKS1B may be heterogeneously expressed in myeloma cells at diagnosis, and current treatments ineffectively eliminate the small populations of CKS1B high-expression myeloma cells, leading to relapse. To test the hypothesis that MM cells with high expression of CKS1B are more drug-resistance and responsible for MM relapse, CKS1B was over-expressed in OCI-MY5 and XG-1 MM cells by lentivirus vector-mediated CKS1B-cDNA transfection (Figure 2A). CKS1B-transfected OCI-MY5 and XG-1 cells were treated with bortezomib (Vel) at a dose of 5 nM for 48 hours. Cell growth and cell survival were examined. Untreated and EV-transfected cells with or without bortezomib served as controls. As shown in Figure 2B & 2C, bortezomib treatment induced significantly less growth inhibition (Figure 2B) and cell death (Figure 2C) in CKS1B-transfected cells compared with EV-transfected controls (*p* <0 .05). Similarly, treatment of doxorubicin (Dox) 100nM (Figure 2D & 2E) and etoposide (Epo) 100nM (Figure 2F & 2G) for 48 hours, induced significantly less growth inhibition and cell death in CKS1B-transfected cells compared with EV-transfected controls (*p* < 0.05).

### CKS1B over-expression activates STAT3 and MEK/ ERK through SKP2 and p27^Kip1^-independent pathways

**Fig. 3:**
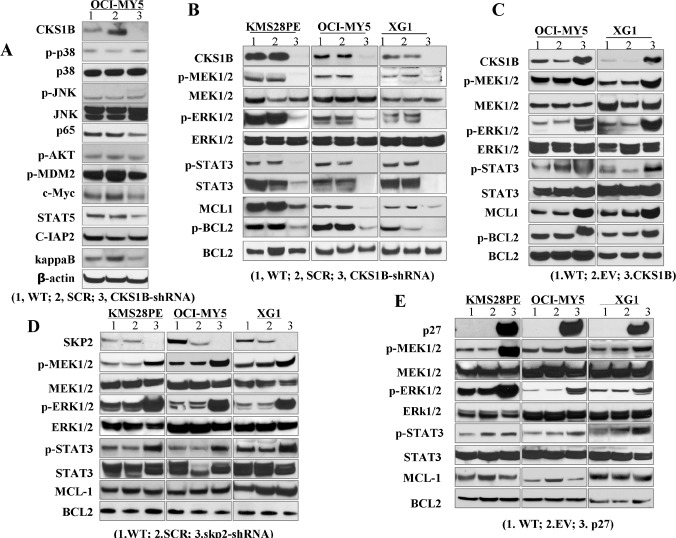
CKS1B-over-expression activates STAT3 and MEK/ERK signaling pathways. CKS1B was knocked down in KMS28PE, OCI-MY5 and XG-1 cells. Cells were cultured for 72 hours and cell lysates were prepared **(A)** CKS1B-induced signaling pathways were screened by western blots in CKS1B-knockdown (CKS1B-sh) OCI-MY5 cells using the indicated antibodies. **(B)** Protein levels of p-STAT3, p-MEK1/2, p-ERK1/2 and p-BCL2 were examined in KMS28PE, OCI-MY5 and XG1 cells after CKS1B-knockdown. Wild-type (WT) and Scramble (SCR)-transfected cells were used as controls and β-actin was used as loading control. **(C-E)** Protein levels of p-STAT3, p-MEK1/2, p-ERK1/2 and p-BCL2 were examined by western blot analysis in CKS1B-transfected, SKP2-silenced, and p27 ^Kip1^-transfected MM cell lines of OCI-MY5 and XG-1 cells, respectively. WT and EV cells were used as controls and β-actin was used as loading control.

To identify these CKS1B-mediated SKP2/p27^Kip1^-independent signaling pathways, western blots were applied to screen for activation of the key signaling pathways which relate to MM cell survival and apoptosis, including MAPK, NF-κB, TP53, PI3K/AKT and STAT3 using OCI-MY5 cells after CKS1B-silencing by specific CKS1B-sh+RNA transfection. Decreased phosphorylated (p)-MEK1/2, p-ERK1/2, p-STAT3 and p-BCL2 levels were detected in CKS1B silenced OCI-MY5 cells compared with wild-type cells and cells transfected with a non-targeting, scramble sequence (SCR) (Figure 3A & 3B). Similar results were also observed in CKS1B silenced KMS28PE and XG-1 cells (Figure 3B). We further examined the alteration of STAT3, MEK/ERK and BCL2 in CKS1B-transfected OCI-MY5 and XG-1 cells. As shown in Figure 3C, increased levels of p-MEK1/2, p-ERK1/2, p-STAT3 and p-BCL2 were observed in CKS1B-transfected OCI-MY5 and XG-1 cells compared with the EV-transfected controls. These results strongly suggest STAT3, MEK/ERK, and BCL2 are the downstream signaling targets of CKS1B.

To examine the functional independence of SKP2 and CKS1B, SKP2-shRNA was used to silence SKP2 in KMS28PE, OCI-MY5, and XG-1 cells and protein levels of p-MEK1/2, p-ERK1/2, p-STAT3, and p-BCL2 in these cells were examined. Wild-type (WT) and SCR-transfected cells were used as controls. As shown in Figure 3D, SKP2-knockdown resulted in an increase rather than a decrease of p-MEK1/2, p-ERK1/2 and p-STAT3 levels in KMS28PE, OCI-MY5 and XG-1 cells, with no remarkable changes in p-BCL2 level. We then examined the functional relationship between p27^Kip1^ and STAT3, MEK/ERK and BCL2 by over-expressing p27^Kip1^ in KMS28PE, OCI-MY5 and XG-1 cells using a lentivirus system. Western blots detected increased levels of p-STAT3, p-MEK1/2 and p-ERK1/2 in p27^Kip1^-transfected myeloma cells with no remarkable effect on p-BCL2 levels (Figure 3E) compared with WT and EV controls.

### Activation of STAT3 is involved in CKS1B-mediated myeloma cell growth and survival

**Fig. 4:**
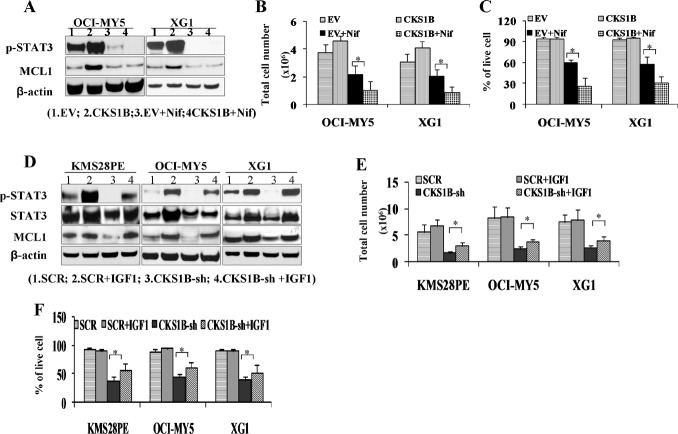
Activation of STAT3 is involved in CKS1B-mediated myeloma cell growth and survival **(A)** 1×10^6^ CKS1B- transfected OCI-MY5 and XG-1 were treated with 10nM Nifuroxazide (Nif) for 48 hours, and cell lysates were prepared. Protein levels of p-STAT3 and MCL1 were analyzed and showed decreased in Nif treated MM cells by Western blots. **(B)** Cell growth and **(C)** cell survival were evaluated. MM cells treated with Nif showed clearly more cell growth inhibition (OCI-MY5: 2.18M vs. 1.04M, and XG-1: 2.07M vs. 0.90M) and more cell death (OCI-MY5: 60% vs. 27%, and XG1: 58% vs. 31) in CKS1B over-expressed cells. Untreated and EV cells with or without Nif treatment were used as controls. **(D)** Western blots showed IGF-1 increased p-STAT3 and MCL1 in CKS1B silenced MM cells. 1×106 OCI-MY5 and XG-1 after CKS1B-knockdown (CKS1B-sh) were treated with 100 ng/mL IGF-1 for 72 hours and p-STAT3 and MCL1 protein levels were analyzed. Untreated CKS1B-sh cells and SCR cells with or without IGF-1 treatment were used as controls. **(E)** Cell growth and **(F)** cell death were evaluated. IGF-1 can partially rescue CKS1B knockout induced cell growth inhibition (KMS28PE: 1.74M vs. 2.88M, OCI-MY5: 2.40M vs. 3.64M, and XG1: 2.60M vs. 3.86M) and apoptosis (KMS28PE: 36% vs. 56%, OCI-MY5: 43% vs. 61%, and XG1: 39% vs. 53%). Untreated cells and SCR cells with or without IGF-1 treatment were used as controls. Results were expressed as Mean + SD of three independent experiments (*p <0 .05).

A specific STAT3 inhibitor Nifuroxazide at the dose of 10 nM for 48 hours was used to treat CKS1B-transfected OCI-MY5 and XG-1 cells. Nifuroxazide treatment decreased p-STAT3 in both EV- and CKS1B-transfected myeloma cells compared with untreated controls by western blot (Figure 4A), and it did not alter the total protein and phosphorylation of MEK/ERK1 (data not shown), confirming its specificity. Nifuroxazide-induced cell growth and cell death were also evaluated in CKS1B-transfected OCI-MY5 and XG-1 cells. Cells were treated with 10 nM Nifuroxazide for 48 hours, Nifuroxazide treatment induced more inhibition of cell growth (Figure 4B) and more increase in cell death (Figure 4C) in CKS1B-transfected cells compared with EV-transfected cells, indicating that myeloma cells with higher CKS1B-expression levels are more sensitive to STAT3 inhibition than cells with lower CKS1B-expression.

Insulin-like growth factor I (IGF-1) is a well-recognized myeloma cell survival factor [10, 11] activating the STAT3 signaling pathway [12]. To further confirm the functional role of STAT3 signaling in CKS1B-mediated myeloma cell growth and survival, CKS1B-silenced KMS28PE, OCI-MY5 and XG-1 cells were treated with IGF-1 (100ng/ml) for 72 hours. Western-blots detected increased p-STAT3 in these CKS1B-silenced myeloma cells compared with untreated, SCR- or CKS1B-shRNA-transfected controls (Figure 4D), confirming activation of STAT3 in IGF-1-treated cells. The effects of IGF-1 treatment on CKS1B-shRNA-induced myeloma cell death and growth inhibition were evaluated. As shown in Figure 4E & 4F, IGF-1 treatment partially abrogated growth inhibition and cell death induced by CKS1B-knockdown (*p* <0 .05).

STAT3 regulates the expression of many target genes involved in cell survival and growth. To investigate whether CKS1B activates STAT3 signaling through regulation of STAT3 target genes, protein levels of cell growth and apoptosis related gene *MCL1* were measured by western blot. [14]. The level of MCL1 was decreased in CKS1B silenced MM cells and increased in CKS1B over-expressing MM cells (Figure 3B & 3C); whereas *MCL1* expression did not show alteration in MM cells manipulated by inhibition of SKP2 and over-expression of p27^Kip1^ (Figure 3D & 3E). Furthermore, MCL1, following STAT3 expression pattern, was significantly increased in IGF1 treated MM cells with inhibited CKS1B expression by CKS1B-shRNA (Figure 4D). These data provide evidence that CKS1B regulates STAT3 signaling and MCL1 transcription.

### Activation of the MEK/ERK signaling pathway is also involved in CKS1B-mediated myeloma cell growth and survival

**Fig. 5:**
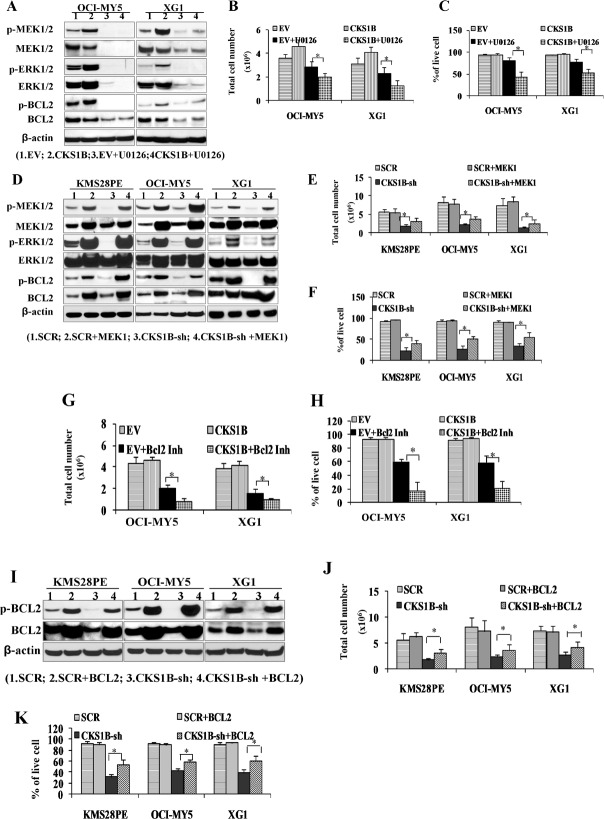
Activation of MEK/ERK signaling pathway is involved in CKS1B-mediated myeloma cell growth and survival **(A)** Western blots showed that MEK inhibitor U0126 inhibited p-MEK, p-ERK, and p-BCL2 expression. 1×10^6^ CKS1B- transfected OCI-MY5 and XG-1 were treated with 10nM U0126 for 48 hours, and p-MEK1/2, p-ERK1/2 and p-BCL2 levels were analyzed. Untreated and EV cells with or without U0126 treatment were used as controls, and β-actin was used as loading control. **(B)** Cell growth and **(C)** cell death were evaluated. MM cells treated with U0126 showed clearly more cell growth inhibition (OCI-MY5: 2.84M vs. 2.01M, and XG1: 2.3M vs. 1.3M) and more cell death (OCI-MY5: 80% vs. 43%, and XG1: 77% vs. 52%) in CKS1B over-expressed cells than in empty vector (EV)-transfected controls. **(D)** Western blots showed p-BCL2 increased in MEK1 over-expressed MM cells. KMS28PE, OCI-MY5 and XG-1 were doubly transfected with CKS1B-shRNA and MEK1-cDNA to knockdown CKS1B and constitutively activate the MEK/ERK signaling pathway in these cells. SCR and CKS1B-sh cells were used as controls, and β-actin was used as loading control. **(E)** Cell growth and **(F)** cell death were evaluated in CKS1B-sh and MEK1 doubly-transfected cells after 72 hours induction. Clearly, MEK1 overexpression can partially rescue CKS1B-shRNA induced cell growth inhibition (KMS28PE: 1.74M vs. 3.10M, OCI-MY5: 2.10M vs. 3.63M, and XG1: 1.20M vs. 2.50M) and cell apoptosis (KMS28PE: 22% vs. 41%, OCI-MY5: 27% vs. 51%, and XG1: 33% vs. 56%). **(G)** Cell growth and **(H)** cell death were evaluated in the treatment of a specific BCL2 inhibitor (BCL2-inh). OCI-MY5 and XG-1 cells transfected with CKS1B showed a stronger cell growth inhibition (OCi-MY5: 1.97M vs. 0.85M, and XG1: 1.53M vs. 0.96M) and cell death (OCI-MY5: 60% vs. 17%, and XG1: 58% vs. 21%) compared with EV- transfected OCI-MY5 and XG-1. **(I)** Western blots showed BCL2 increased in OCI-MY5 and XG-1doubly transfected with CKS1B-shRNA and BCL2-cDNA. **(J)** Cell growth and **(K)** cell death were evaluated in CKS1B-sh and BCL2 doubly-transfected cells after 72 hours induction. Over-expression of BCL2 in MM cells can partially rescue CKS1B knockout induced cell growth inhibition (KMS28PE: 1.74M vs. 3.10M, OCI-MY5: 2.40M vs. 3.63M, and XG1: 2.60M vs. 4.20M) and apoptosis (KMS28PE: 32% vs. 55%, OCI-MY5: 43% vs. 59%, and XG1: 39% vs. 61%). Scramble (SCR) and CKS1B-sh cells served as controls. Results were expressed as Mean ± SD of three independent experiments (*p <0 .05).

A specific MEK inhibitor U0126 was used to treat CKS1B-transfected OCI-MY5 and XG-1 cells for 48 hours at the dose of 10 μM. Cell lysates were prepared and subjected to western analysis using antibodies recognizing p-MEK1/2 and p-ERK1/2 proteins. U0126 decreased p-MEK1/2 and p-ERK1/2 in both EV- and CKS1B-transfected myeloma cells (Figure 5A). However, U0126 also shown to decrease p-STAT3 indicating this compound is not specific inhibitor of MEK/ERK.. Interestingly, U0126 treatment down-regulated BCL2 in myeloma cells (Figure 5A), suggesting that BCL2 represents a downstream target of the MEK/ERK signaling pathway. We also evaluated U0126-induced cell growth and cell viability in CKS1B-transfected OCI-MY5 and XG-1 cells. Cells were treated with 10 μM U0126 for 48 hours, U0126-treatment induced more cell growth inhibition (Figure 5B) and cell death (Figure 5C) in CKS1B-transfected cells than those in EV-transfected cells (*p* < 0.05).

Since inhibitors of MEK/ERK signaling pathway may induce some non-specific effects on myeloma cell growth and survival, we constitutively activated the MEK/ ERK signaling pathway by lentivirus-vector-mediated MEK1-cDNA transfection in CKS1B-silenced KMS28PE, OCI-MY5 and XG1 cells. Western blots showed a larger increase of p-MEK1/2 and p-ERK1/2 levels in MEK1-cDNA and CKS1B-shRNA double-transfected cells compared with SCR- and CKS1B-transfected controls (Figure 5D). MEK1-transfection increased protein levels of p-BCL2 in CKS1B-silenced cells (Figure 5D), indicating that MEK1-transfection could overcome CKS1B-knockdown-mediated inhibition of BCL2 signaling pathway [13]. Although MEK1-cDNA and CKS1B-shRNA doubly-transfected cells clearly showed increased cell death and growth inhibition compared with SCR-transfected control cells (Figure 5E & 5F, *p* < 0.05), these double-transfected cells exhibited significantly less cell death and growth inhibition than CKS1B-silenced myeloma cells (Figure 5E & 5F, *p* <0 .05), indicating that MEK1-transfection partially abrogated cell death and growth inhibition induced by CKS1B-knockdown.

Our results indicate that BCL2 is a downstream target of the CKS1B and MEK/ERK signaling pathway. Therefore, a specific BCL2 inhibitor (2,9-Dimethoxy-11,12-dihydrodibenzo[c,g][1,2]-diazocine 5,6-dioxide and 5,5'-Dimethoxy-2,2'-dinitrosobenzyl) at the dose of 10 nM for 48 hours was used to treat CKS1B-transfected OCI-MY5 and XG-1 cells. Treatment with this BCL2-inhibitor resulted in significantly more cell growth inhibition (Figure 5G) and cell death (Figure 5H) in CKS1B-transfected cells compared with EV-transfected cells, indicating that myeloma cells with higher CKS1B-expression are more sensitive to BCL2 inhibition than cells with lower CKS1B-expression. We subsequently over-expressed BCL2 by lentivirus-mediated BCL2 cDNA transfection in CKS1B-silenced KMS28PE, OCI-MY5 and XG-1 cells. Cells were cultured for 4 days and western blots confirmed increased p-BCL2 levels in BCL2 cDNA and CKS1B-shRNA doubly-transfected cells (Figure 5I). The effects of BCL2-transfection on CKS1B-shRNA induced myeloma cell growth inhibition and death were evaluated. As shown in Figure 5J & 5K, although BCL2-cDNA and CKS1B-shRNA doubly-transfected cells clearly showed cell growth inhibition and death compared with SCR-transfected control cells (*p* <0 .05), BCL2 transfection partially abrogated myeloma cell growth inhibition and death induced by CKS1B-silencing (Figure 5J & 5K, *p* < 0.05).

### Combination of STAT3 and MEK1 inhibitors induced synergistic cytotoxicity on myeloma cells with high CKS1B expression

**Fig. 6:**
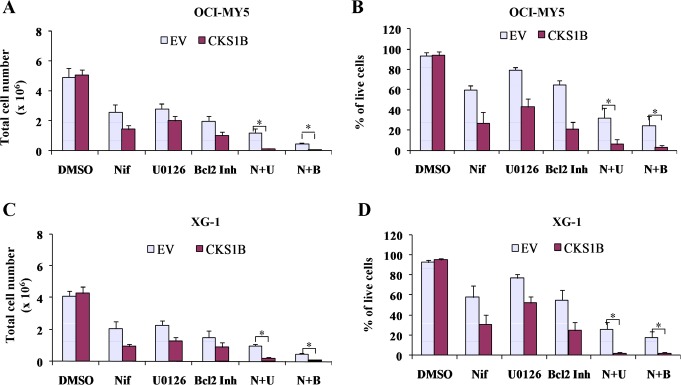
Combinations of STAT3 and MEK inhibitors induced synergistic cytotoxicities on CKS1B-transfected myeloma cells **(A)** Cell growth and **(B)** cell death were evaluated in CKS1B-transfected OCI-MY5 treated with MEK1 inhibitor U0126 (10 μM) plus STAT3 inhibitor Nifuroxazide (10 nM) (N+U Inh) and Nifuroxazide plus BCL2 inhibitor (10 nM) (N+B Inh) for 24 hours. The total cell numbers and cell viability between empty vector (EV) vs. CKS1B-cDNA transfected OCI-MY5 cell line are 4.85M vs. 5.11M and 92% vs. 90% in DMSO, 3.10M vs. 1.61M and 61% vs. 35% in Nif, 3.46M vs. 2.10M and 78% vs. 53% in U0126, 2.87M vs. 0.85M and 64% vs. 32% in BCL2 Inh, 0.90M vs. 0.17M and 39% vs. 14% in N + U, and 0.36M vs. 0.07M and 29% vs. 11% in N + B, respectively. **(C)** Cell growth and **(D)** cell death were evaluated in CKS1B-transfected XG1 treated with N+U Inh and N+B Inh for 24 hours. The total cell numbers and cell viability between empty vector (EV) vs. CKS1B-cDNA transfected XG1 cell line are 4.12M vs. 4.30M and 90% vs. 92% in DMSO, 2.17M vs. 1.64M and 62% vs. 35% in Nif, 2.63M vs. 1.83M and 78% vs. 52% in U0126, 2.03M vs. 0.96M and 65% vs. 32% in BCL2 Inh, 0.97M vs. 0.20M and 33% vs. 2% in N + U, and 0.58M vs. 0.09M and 27% vs. 3% in N + B, respectively. The Combination Indices values of EV- and CKS1B-transfected OCI-MY5 and XG-1 cells treated with combination of STAT3 and MEK1 inhibitors are 0.44 and 0.30, and 0.69 and 0.27, respectively; while these cells treated with combination of STAT3 and BCL2 inhibitors are 0.37 and 0.27, and 0.50 and 0.26, respectively. Both combinations showed synergistic effects in inhibition MM cell growth and induction MM cell apoptosis. Untreated cells and EV-transfected cells with or without treatment served as controls. Results were expressed as Mean ± SD of three independent experiments (*p < 0.05 and **p < 0.01).

Since the STAT3 and MEK/ERK signaling pathways were both involved in CKS1B-induced myeloma cell growth and survival, a combined targeting of these signaling pathways might induce synergistic cytotoxicity in myeloma cells. CKS1B-transfected OCI-MY5 cells were treated with combination of STAT3 and MEK1 inhibitors (i.e. U0126 [10 μM] plus Nifuroxazide [10 nM]), or combination of STAT3 and BCL2 inhibitors (i.e. U0126 [10 μM] plus BCL2 inhibitor [10 nM]) for 48 hours. EV-transfected cells treated with the same combinations were used as controls. Untreated CKS1B- and EV-transfected cells also served as controls. As shown in Figure 6A & 6B, the combinations of the STAT3 with MEK1 and the STAT3 with BCL2 inhibitors induced significant less cell growth (Figure 6A) and more cell death (Figure 6B) in CKS1B-transfected OCI-MY5 compared with EV-transfected control cells (*p* <0.01). CKS1B-transfected XG-1 cells were also treated with the same combinations for 48 hours. Similar to the CKS1B-transfected OCI-MY5 cells, the combinations of the STAT3 with MEK1 and STAT3 with BCL2 inhibitors induced significant less cell growth (Figure 6C) and more cell death (Figure 6D) in CKS1B-transfected XG-1 cells compared with EV-transfected control cells (*p* < 0.01). Combination Indices (CI) were also calculated according to the method that was developed by Chou and Talalay [14]. The CI values of EV- and CKS1B-transfected OCI-MY5 and XG-1 cells treated with combination of STAT3 and MEK1 inhibitors are 0.44 and 0.30, and 0.69 and 0.27, respectively; while these cells treated with combination of STAT3 and BCL2 inhibitors are 0.37 and 0.27, and 0.50 and 0.26, respectively. The lower CI values were shown in the CKS1B-transfected cells, which means the synergism of the combinations are stronger in the CKS1B-transfected cells compared with the EV-transfected cells. Our results demonstrated that these combinations exhibit synergistic effect and are very toxic to myeloma cells even when they do not over-express CKS1B, but myeloma cells with higher CKS1B-expression are more sensitive to combinations therapy with STAT3 and MEK/ERK inhibitors than cells with lower CKS1B-expression.

## DISCUSSION

Expression of CKS1B, which maps to a previously defined amplicon at 1q21 and regulates SCF^Skp2^-mediated ubiquitination and proteolysis of the CDK inhibitor p27^Kip1^, was significantly overexpressed in newly diagnosed myeloma patients with a short survival [3, 4]. In this study, we showed that high expression of CKS1B does also predict post-relapse survival. Our study showed a marked increase of CKS1B expression in relapsed MMs, which provides molecular evidence of a more drug-resistant disease.

The aim of the present study was to further clarify the functional role of CKS1B in myeloma cell survival and drug-resistance by investigating CKS1B-induced SKP2- and p27^Kip1^-independent signaling pathways. Our results show that forced-expression of CKS1B in MM cells induces multidrug-resistance, providing direct evidence for the crucial role of CKS1B in myeloma progression. Using western blots, we found that over-expression of CKS1B stimulates STAT3 and MEK/ERK, whereas SKP2 knockdown or p27^Kip1^ over-expression activated rather than suppressed STAT3 and MEK/ERK pathways, suggesting that SKP2 over-expression or p27^Kip1^ inhibition exerted the opposite effect of CKS1B over-expression on STAT3 and MEK/ERK. We also showed that BCL2 is a downstream target of CKS1B- induced MEK/ERK signaling. Moreover, stimulation of STAT3 and MEK/ERK/BCL2 signaling pathways partially abrogated MM cell death and growth inhibition induced by CKS1B-knockdown.

Targeting STAT3 and MEK/ERK/BCL2 activity by specific inhibitors resulted in significant MM cell death and growth inhibition and their combinations had a synergistic cytotoxic effect on myeloma cells. Thus, our findings clarify the SKP2/ p27^Kip1^-independent mechanisms of CKS1B activity in maintaining myeloma cell growth and survival, and also provide a rationale for specifically targeting STAT3 and MEK/ERK/BCL2 in aggressive CKS1B-overexpressing MM.

CKS1B is one of the 70 high-risk signature genes associated with poor outcome in newly diagnosed myeloma [3]. Elevated expression of *CKS1B* is a predictor of poor prognosis and aggressive disease in many other malignancies [15-18]. Recently, Westbrook et al reported that Cks1 mRNA and protein were significantly up-regulated in transgenic mice with mammary tumors initiated by erbB2, c-myc and polyoma middle-T (PyMT) [19]. Consistent with our current report, mammary tumor cells derived from both transgenic mice and cell lines with high expression of Cks1 failed to reveal a decrease of p27^Kip1^; in fact p27^Kip1^ levels were slightly higher in mammary tumors initiated by erbB2, PyMT and MNU [19]. We previously showed that CKS1B is essential for myeloma cell growth and survival by using gene knockdown [4]. In the present study, forced expression of CKS1B was found to promote multidrug-resistance. Based on our results, it is reasonable to speculate that high CKS1B expression will also result in increased drug resistance in other malignancies.

We have reported that dysregulation of expression of one of the three type D cyclins is likely to be an initiating genetic lesion in most myelomas and monoclonal gammopathy of undetermined significance (MGUS) [20, 21]. Dysregulation of type D cyclins is a common event in cancer that contributes to tumorigenesis by promoting hyper-phosphorylation of pRB1 and activation of E2F target genes, which are important in promoting the transition of cells through early G1-phase to S-phase [22-24]. RNA interference of *CKS1B* mRNA in myeloma cells led to reduced CKS1B mRNA and protein, an accumulation of p27^Kip1^, and profound growth inhibition [4]. Based on these observations, we conclude that while activation of a type D cyclin is an early initiating event, *CKS1B* over-expression is a progression event; both events are required for the loss of early and late G_1_- to S-phase cell cycle checkpoints and to establish an aggressive clinical phenotype. Recent studies indicate that Cks1 is required not only for G1/S, but also for G2/M transition [25-27]. Knockdown of Cks1 in mouse embryonic fibroblasts or Hela cells results in cell cycle arrest in G2 and blocks cell proliferation. Over-expression of cyclin B1 rescues this cell cycle arrest [27]. Thus, these observations further demonstrate that CKS1B functions indeed at multiple levels.

In this study, we found that CKS1B-knockdown inhibited and CKS1B-overexpression activated STAT3 and MEK/ERK signaling pathways, demonstrating that STAT3 and MEK/ERK signaling pathways are the CKS1B downstream signaling pathways, independent of SKP2/p27^Kip1^. Further investigation showed that BCL2 is also a downstream target of MEK/ERK signaling pathway. Our results are consistent with the recent investigations which showed that CKS1B stimulated MEK/ERK signaling pathway in the breast cancer cells [28, 29]. Our findings explain why SKP2 knockdown or p27^Kip1^ over-expression induces less myeloma cell growth inhibition and cell death than CKS1B knockdown [4]. Myeloma cells with forced CKS1B expression are more sensitive to treatment with specific inhibitors targeting these signaling pathways, while stimulation of these signaling pathways partially abrogates myeloma cell death and growth inhibition, induced by CKS1B knockdown. Thus, it is reasonable to conclude that CKS1B mediates myeloma cell growth and drug-resistance through activation of STAT3 and MEK/ERK/BCL2 signaling pathways.

In conclusion, this study further demonstrates that CKS1B plays a crucial role in MM cell growth and survival and for the first time provides direct evidence for the crucial role of CKS1B in myeloma multidrug-resistance. We identified STAT3 and MEK/ERK/BCL2 as CKS1B-downstream signaling pathways; and thereby provided targets for the development of new therapeutic approaches for CKS1B over-expressing myeloma and other tumor malignancies. Since CKS1B over-expression and 1q21 gains have been observed in many other malignancies and it is well-documented that JAK/STAT3 and MEK/ERK/BCL2 signaling pathways play a crucial role in tumor cell survival, drug-resistance and cancer progression [30-38], our findings might have broad applications for cancer treatment. Further preclinical *in-vivo* animal studies are needed to confirm our *in-vitro* findings.

## MATERIALS AND METHODS

### Study subjects and gene Expression Profiling (GEP)

51 paired myeloma samples collected at baseline and at early relapse were obtained from newly diagnosed MM patients who were treated on the National Institutes of Health-sponsored clinical trials UARK 98-026 (Total Therapy 2 [TT2], n = 668) [39, 40]. This protocol used induction regimens followed by melphalan-based tandem autotransplantation, consolidation chemotherapy, and maintenance treatment. All subjects provided written informed consent, acknowledging the investigational nature of the protocol and the availability of other treatment options, as required by the Institutional Review Board and the Food and Drug Administration and in line with the Helsinki Declaration.

The plasma cells were isolated by automated CD138 immunomagnetic bead selection (Miltenyi Biotec). CD38/ CD45 flow cytometry determined that plasma cell purity was routinely 85% or greater [41, 42]. 51 myeloma patients with paired GEPs from U133 Plus2 Affymetrix microarray at baseline and at relapse were used in this study. All GEP data included in the study were reported in our previous publications [29, 42, 43]. Paired Student *t* test were used to compare *CKS1B* expression between baseline and relapse samples. The Kaplan-Meier method was used to estimate post-relapse survival.

### Human myeloma cell lines and reagents

Myeloma cell lines KMS28PE,OCI-MY5, and XG1 were cultured in RPMI 1640 containing 10% heat-inactivated fetal bovine serum (FBS), 2 mM L-glutamine (Gibco, Grand Island, NY), penicillin (100 U/mL), and streptomycin (100 μg/mL) in a humidified incubator at 37°C in 5% CO_2_.

Bortezomib was obtained from Millennium Pharmaceuticals (Cambridge, MA). Doxorubicin and etoposide were purchased from Sigma (Louis, MO). The MEK inhibitor U0126 was obtained from Promega (Madison, WI); the STAT3 inhibitor Nifuroxazide was purchased from Sigma and the Bcl2 inhibitor (2,9-Dimethoxy-11,12-dihydrodibenzo[c,g][1,2]-diazocine 5,6-dioxide and 5,5'-Dimethoxy-2,2'-dinitrosobenzyl) was purchased from Emd biosciences (San Diego, CA). The BCL2 inhibitor is not a specific compound to target BCL2, it inhibits the activity of both BCL2 and BCL-xl. These inhibitors were dissolved in DMSO (Sigma), and the final concentration of DMSO in cultures was 0.05%. IGF-1 was obtained from R&D (Minneapolis, MN).

### Specific gene silencing or overexpressing by lentivirus expression vector system

The lentiviral system in this study was kindly gifted from Dr. Didier Trono (National Center for Competence in Research, Switzerland). This system includes the lentiviral vector pLVTH, the envelop plasmid pMD2G, and the packaging plasmid pCMV-dR8.74. Specific gene knockdown was previously described [4, 29]. Similar methods are used for gene over-expression by this system. Recombinant lentivirus was produced by transient transfection of 293T cells following a standard protocol [44]. Briefly, crude virus was concentrated by ultracentrifugation at 9 000 g for 90 minutes. Viral titers were determined by measuring the amount of HIV-1 p24 antigen by enzyme-linked immunosorbent assay (NEN Life Sciences, Boston, MA). A 99% transduction efficiency of MM cells was achieved with 3000 ng lentiviral p24 particles/10^6^ cells. Myeloma cells with CKS1B-knockdown, SKP2-knockdown or p27^Kip1^ over-expression were described previously [4]. Primers for cDNA clones (CKS1B forward, 5'-CGA TCA TGT CGC ACA AAC A-3', and reverse, 5'-GCC AGC TTC ATT TCT TTG GT-3'; MEK1 forward,5'- GTC CAA AAT GCC CAA GAA GA-3', and reverse, 5'-CAA ACA CTT AGA CGC CAG CA-3'; and BCL2 forward, 5'-GTC CAA GAA TGC AAA GCA CA-3', and reverse, 5'-AGC CTG CTT TGT TTC AT-3') were purchased from Invitrogen (Carlsbad, CA). Recombinant lentiviruses were produced by transient transfection of 293T cells following a standard protocol [44]. Efficiency of viral transfection was determined by counting the number of GFP expressing cells using flow cytometry and the transduction efficiency of MM cells was above 95%.

### Treatment of CKS1B-overexpressing MM cells with chemotherapeutic drugs

To determine the role of CKS1B in MM drug-resistance, OCI-MY5 and XG-1 cells were transfected with CKS1B-cDNA to overexpress CKS1B in these cell lines by lentivirus expression vector system. 1×10^6^ cells were treated with bortezomib at 5 nM for 48 hours in the cultures. Cell growth was evaluated by cell counting with a hemocytometer, and dead cells were determined by trypan blue staining, from which dead-cell fraction was calculated. Untreated cells and empty-vector (EV) transfected cells with or without drug-treatment were used as controls. Similar experiments were performed for doxorubicin (100 nM) and etoposide (100 nM), and cells were treated for 48 hours.

### Western Blots

Western blots were performed to examine the protein levels in MM cells as previously described [45, 46]. CKS1B antibodies were purchased from Invitrogen (Carlsbad, CA) and all other primary antibodies were purchased from Cell Signaling Technology (Beverly, MA). β-actin was used to normalize the amount of protein in each sample.

### Statistical Analysis

One-way ANOVA (≥ 3groups) and Student *t* test (=2 groups) were used to compare various experimental groups. Significance was set at *P* < 0.05.
